# Assessing Efficiency of Urban Land Utilisation under Environmental Constraints in Yangtze River Delta, China

**DOI:** 10.3390/ijerph182312634

**Published:** 2021-11-30

**Authors:** Yue Zhou, Yi Chen, Yi Hu

**Affiliations:** School of Geography and Ocean Science, Nanjing University, Nanjing 210023, China; zhouyue092798@163.com (Y.Z.); huyi0234@163.com (Y.H.)

**Keywords:** SE-SBM, construction land-use efficiency, environmental constraints, Yangtze River Delta, China

## Abstract

Measuring the efficiency of construction land utilisation is important for optimising the allocation of regional resources and guiding the sustainable development of the regional society and economy. Based on municipal panel data on urban land use from 2009 to 2017 from a municipal perspective, this research built a slacks-based measure of a super-efficiency model (SE-SBM) to evaluate the temporal and spatial differentiation characteristics of the construction land-use efficiency of 41 cities in the Yangtze River Delta. Following this, the driving force of construction land efficiency was calculated using the Malmquist–Luenberger index. Finally, the entropy-weight TOPSIS (technique for order preference by similarity to ideal solution) model and the k-means clustering method were applied to evaluate an input–output model of the cities. The main conclusions are as follows: (1) The construction land efficiency of the Yangtze River Delta remains at a low level and presents a spatial differentiation pattern, with the efficiency being higher in the east and lower in the west. Due to undesired outputs, the mean value has dropped by 4.67%, and the regional imbalance has decreased. (2) The degree of efficiency loss is significantly positively correlated with the intensity of urban pollution emissions—the higher the pollution emissions, the greater the efficiency loss. (3) The total factor productivity of urban construction land is mainly driven by technological progress, while the promotion of technical efficiency is low and unstable. (4) The evaluation of construction land efficiency must include resource allocation or pollution emission factors to scientifically measure the input–output level. These research results will help to formulate reasonable land-use countermeasures.

## 1. Introduction

The efficient use of urban construction land, the site of economic and social activities [1], is essential for the sustainable development of the urban economy [2,3]. There are many ways to measure whether urban land has been used efficiently, among which measuring the land-use efficiency is the most direct, effective, and universal. More importantly, the efficiency of land use is not only able to reflect the allocation of land and space resources but is also related to the scientific exploration of human settlements and human well-being. There has been abundant research on land-use efficiency evaluations, both nationally and internationally [4,5]. 

Regarding research approaches, scholars mostly use the traditional data envelopment analysis (DEA) model, the Cobb–Douglas production function, and stochastic frontier analysis, among other methods, to measure the land inputs and outputs of an entire country [6,7], regions [8], provinces [9], cities [10,11,12], urban agglomerations [13,14], or counties [15]. In the past, scholars mostly began from an economic perspective and measured the land-use efficiency using the single economic output of the land [16]. Chen used the traditional DEA model to measure the efficiency of industrial land use in China’s resource-based cities, revealing regional differences [17]. Liu used an extended Cobb–Douglas production function to determine that the efficiency of construction land allocation in China needed to be further improved, while the intensive use of land resources was also necessary [18]. Gui introduced stochastic frontier analysis (SFA) to determine that urban land-use efficiency in the Yangtze River Economic Zone showed a significant growth trend with a cumulative growth rate of 54.07% [19]. 

However, in recent years, as the green “people-oriented” development concept has become the social consensus, relevant research has shifted its perspective from the economic benefits of land use to the ecological benefits [20] and has begun to focus on the spatial-distribution characteristics of comprehensive benefits [21], such as economic, social, and ecological benefits, within the region. Generally speaking, scholars characterize the concept of greenness by considering undesired outputs (e.g., wastewater and carbon emissions), which not only reflects the ecological connotations of urban land use but also highlights the coordination relationship between humans and land [22]. Yu attempted to incorporate ecological factors into the urban-agglomeration land-use efficiency evaluation index system and found that the efficiency of urban-agglomeration construction land under ecological constraints was significantly reduced, which is consistent with the actual situation [23]. Liang believes that only a measurement factor framework containing undesired outputs can be used to obtain scientific results for urban land-use efficiency and contribute to coordinated urban development [24]. Liu adopted a one-stage SFA model to reveal the potential to improve urban land-use efficiency [25]. On the basis of explaining the connotations of land-use efficiency, Hu constructed an evaluation index system for comprehensive land-use efficiency. The analysis found that the comprehensive benefits of land use within the Jiangsu Province differed significantly, and the gradient structure was more obvious, with the efficiency generally decreasing from southern Jiangsu to northern Jiangsu [26]. In addition, many scholars usually use Malmquist index, Tobit model, panel threshold model, and other spatial measurement methods to study the change mechanism behind land use efficiency, as well as the corresponding optimal allocation and intensive use of urban land [27,28,29]. 

These studies provide a reference for formulating countermeasures for the efficient use of urban construction land resources and the optimization of the corresponding industrial layouts. Conversely, they provide reference for countermeasures to promote the healthy coupling of high-quality economic development and the ecological environment. Zhao applied an extended STIRPAT (Stochastic Impacts by Regression on Population, Affluence and Technology) model to explore the relationship between new-type urbanization and land eco-efficiency. The evidence revealed that the relationship follows an N-shaped curve [30]. According to a Finnish study, at the macroeconomic level, the domestic use of biomass per unit of value added decreased (−2.2%/a) as the amount of human appropriation of net primary productivity (HANPP) per unit of biomass decreased (−1.1%/a), reflecting increased economic efficiency in land use [31]. Some scholars have also found that adding a HSR (High-Speed Rail) route will increase urban land-use efficiency by 0.012 in the case that the city has opened HSR [32]. Overall, the existing results provide a useful reference for further research on the use efficiency of urban construction land. However, scholars have mainly discussed the temporal and spatial characteristics and evolutionary dynamics of efficiency itself, and insufficient attention has been paid to quantitative research on the relationship between efficiency and input–output factors. Therefore, additional efforts are needed in the future to make up for the lack of research in this field.

Since the reform and opening up, land use in the Yangtze River Delta has been characterized by a spatial expansion of construction land, a sharp decline of high-quality arable land resources, and an increase in environmental pollution, which has limited urban development to a certain extent. Especially after the 2008 financial crisis, the Yangtze River Delta is facing more unstable factors, and economic development has entered a new growth cycle, which puts forward higher requirements for the coupling coordination between economic development, social progress and ecological protection. A scientific evaluation of construction land-use efficiency is a key step for sustainable regional development including economy, society and environment. However, there are few relevant studies on this area, which is not conducive to promoting the long-term development of the region. In conclusion, quantitative studies to explain the utilization efficiency of land input in economic growth in this period is warranted, which is also of important reference significance for the formulation and implementation of the new round of *Three-year Action Plan for The Integrated Development of the Yangtze River Delta region (2021–2023)*. 

The aim of this study was to use a slacks-based measure of super-efficiency model (SE-SBM) to quantify the current status of construction land-use efficiency in 41 cities in the Yangtze River Delta, use the Malmquist–Luenberger index (ML) to identify the key driving forces of the evolution of urban construction land-use efficiency; use the entropy-weight TOPSIS model to explore the specific correlation between the city’s input, output, and pollution emissions; and use the k-means clustering method to judge the urban construction land-use mode. Through the above calculations, the study explored the unevenness of the construction land-utilisation efficiency, technological innovation level, and management systems and mechanism levels of cities in the Yangtze River Delta region; identified the problems existing in the construction land-utilisation process of each city; and tried to identify the improvement directions of different types of cities in the process of future development. In the context of global integration, the subsequent development of cities and creative cooperation between cities require new developmental increments. Construction land has always been an important engine for regional economic and social development and an essential place for high-quality development. By providing relevant suggestions for the optimization and management of regional construction land, this research can help the Yangtze River Delta to form a truly stronger, larger, and more concentrated world-class integrated urban-development area, and help it to stabilize its strategic position in the overall situation of national modernization and all-round opening to the outside world. 

## 2. Data Sources and Research Methods

### 2.1. Overview of the Study Area

**Figure 1:**
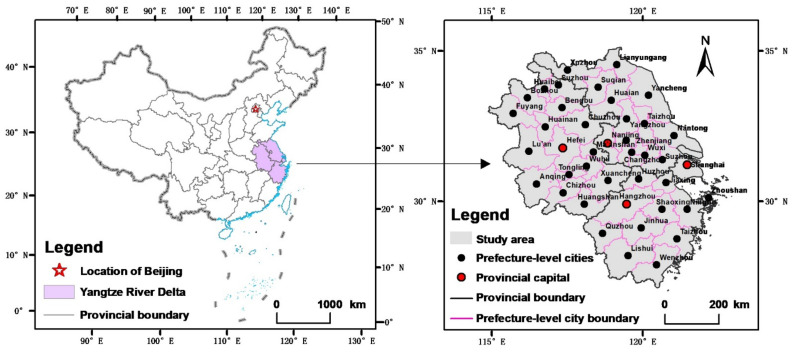
The geographical location and administrative divisions of study area.

The Yangtze River Delta includes three provinces and one city, namely, Jiangsu, Zhejiang, Anhui and Shanghai, respectively, with a total of 41 prefecture-level cities (Figure 1). The *Outline of integrated development of the Yangtze River Delta* [33] pointed out that the Yangtze River Delta is experiencing strong and active growth in the new era and is a new focus of reform and opening. At the end of 2017, the total permanent population of the region was 224 million, the per capita gross domestic product (GDP) was CNY 88,600, and the urbanisation level reached 66.35%, together giving the region a leading position within the country. Further, the economic and social development have expanded rapidly, along with the construction land in the region, which increased from 5.3063 million hectares in 2009 to 6.1865 million hectares in 2017. The average annual growth of construction land during this period was 1.94%, which significantly exceeded the national average. Furthermore, the expansion of construction land led to insufficient arable land reserve resources. At the end of 2017, the per capita arable land area in the Yangtze River Delta was less than 0.072 hm^2^, which was far lower than the national average of 0.097 hm^2^. In addition, ecological and environmental problems, such as water resources, solid waste, and air pollution in the region have become increasingly severe. Regional construction mostly relies on natural resource endowments, and the development model is restricted by traditional thinking. Although the benefits are good, the cost and the emissions are high, and they no longer meet the strategic requirements of ecological civilisation construction. Therefore, promoting the green development of construction land and tapping into the potential of construction land utilisation have become important tasks that must be solved during the urbanisation process of the Yangtze River Delta.

### 2.2. Data Source

This study uses land use and socioeconomic panel data on 41 cities in the Yangtze River Delta region from 2009–2017 as a sample. In the data, construction land includes the following three types of land: urban-rural construction land, land for transportation and water conservancy, and other construction land. The data come from the Natural Resources Department’s land-use change survey data, and the socioeconomic data come from the China City Statistical Yearbook (2010–2018) [34] and the provincial and municipal statistical yearbooks (2010–2018) [35,36,37,38]. In addition, during the study period, the administrative divisions of Chaohu, Tongling, Anqing, Lu’an and Huainan were adjusted. This study uses the new administrative division as the benchmark and decomposes and merges the corresponding indicators according to the adjustment of the administrative division.

### 2.3. Research Methods

#### 2.3.1. The Research Design of the Paper

**Figure 2:**
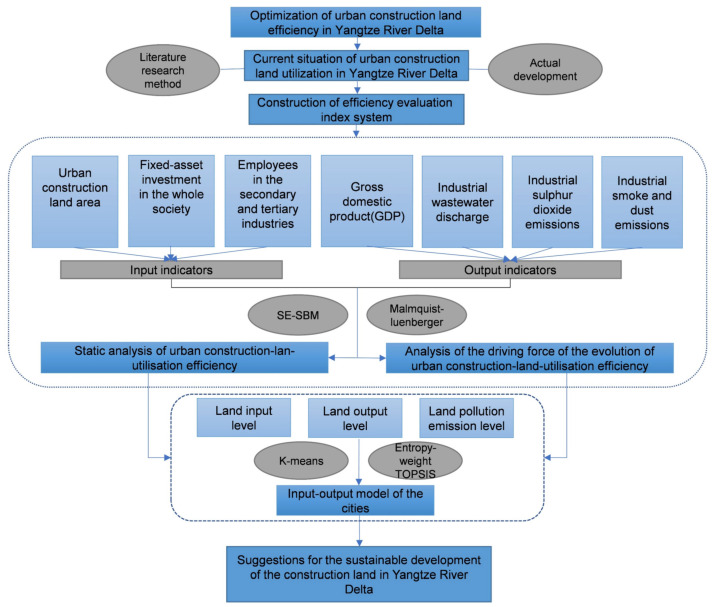
Flowchart of construction land-use optimization.

First, based on understanding the current status of construction land use in the Yangtze River Delta region, combined with literature reading, this study screened the input-output evaluation index system for construction land use in line with the actual development of the Yangtze River Delta from the perspectives of land, capital, labour, and output. Second, the article uses the SE-SBM model and the Malmquist–Luenberger model to calculate the static and dynamic efficiency of construction land use in cities in the Yangtze River Delta. Third, we used the entropy-weight TOPSIS method and K-means clustering method to determine the land use types of 41 cities based on the input, output, and pollution emission levels of each city. Finally, combining efficiency characteristics and input-output types, we put forward policy recommendations for optimizing construction land-utilization efficiency. The research design of this article is shown in Figure 2.

#### 2.3.2. Construction of the Evaluation Index System

According to the current situation of construction land utilisation in the Yangtze River Delta region, by referring to existing studies [39,40,41] and based on the principles of a scientific, systematic, and representative selection of indicators, this work constructed an input–output evaluation index system for construction land in the Yangtze River Delta region (Table 1). Amongst the indicators, the input indicators include land, capital, and labour, which are characterised by the area of urban construction land, fixed-asset investments of the whole society, and employment numbers in the secondary and tertiary industries, respectively. The output indicators include the expected economic output and undesired environmental outputs, which are denoted by the gross regional product and the total discharge of industrial wastewater, exhaust gas, and dust waste. In addition, the entropy-weight TOPSIS method was used to synthesise the three types of waste data to characterise a comprehensive pollution-emission index before the calculations.

#### 2.3.3. SE-SBM with Undesirable Output Model

Data envelopment analysis (DEA) [42] models are widely used to evaluate the efficiency status of decision-making units (DEA uses a decision-making unit (DMU) as its measurement object of efficiency). As it does not require there to be a lack of high correlation (collinearity) between the input indicators and output indicators, and there is no need to estimate parameters or weight assumptions in advance, DEA is especially suitable for systems with multiple inputs and multiple outputs. In 1978, Charnes, Cooper, and Rhodes created the first DEA model, called the CCR model, based on constant returns to scale (CRS). Its basic concept is to take one DMU as an evaluated unit and create an evaluation group with other DMUs, establish a mathematical model corresponding to the problem, and comprehensively analyse the relative efficiency (within the interval of (0, 1)) by solving the results of the model. Then, the production possibility set (PPS) and the production frontier (PF) are determined. According to the distance between the DMUs and the PF, we can determine whether the DMUs are DEA-effective or not. Then, we order the evaluation results. It should be noted that, in the DEA theory, the input and output vectors of the production activities of the decision-making unit are combined into the PPS. The PF is an “envelope surface” formed by the combination of input and output used to achieve maximum efficiency in the PPS. A DMU can be categorized into one of two states: effective or invalid. To judge whether a DMU is DEA effective is, essentially, to judge whether it falls into the PF of the PPS. 

In the traditional DEA model, when multiple DMU are evaluated as effective, the maximum efficiency value obtained by the DEA model is 1, namely, the effective DMU efficiency value is the same. Therefore, The efficiency of these effective DMUs cannot be further distinguished.In addition, in the traditional DEA model, the weight coefficient used to calculate the efficiency value is set in a specific range that is most beneficial to the evaluated unit (maximizing its efficiency value), making it easy to exaggerate advantages and avoid shortcomings. As the traditional DEA model cannot evaluate the efficiency most reasonably and is unable to reorder multiple DMUs with an efficiency value of 1 [43]. Tone [44] further revised the DEA model and proposed the SE-SBM model to make up for the abovementioned defect. Based on the SE-SBM model with undesired outputs (SE-SBM-UN), this study measured the construction land-utilisation efficiency of cities in the Yangtze River Delta. The non-oriented CRS SE-SBM-UN model is expressed as follows:

(1) $$\min p=\frac{1+\frac{1}{e}\sum_{i=1}^{e}\frac{s_{i}^{-}}{a_{ik}}}{1-\frac{1}{r_{1}+r_{2}}\left(\sum_{r=1}^{r_{1}}\frac{s_{r}^{g^{+}}}{b_{rk}^{g}}+\sum_{t=1}^{r_{2}}\frac{s_{t}^{h^{-}}}{b_{tk}^{h}}\right)}\;$$

(2) $$s.t.\;\sum_{j=1,j\neq k}^{u}a_{ij}\lambda_{j}-s_{i}^{-}\leq a_{ik}$$

(3) $$\sum_{j=1,j\neq k}^{u}b_{rj}^{h}-s_{t}^{h^{-}}\leq b_{tk}^{h}$$

(4) $$\sum_{j=1,j\neq k}^{u}b_{tj}^{h}\lambda_{j}+s_{r}^{g^{+}}\geq b_{rk}^{g}$$

(5) $$\lambda ,s^{-},s^{g},s^{h}\geq 0$$

(6) $$i=1,\cdots ,e;r=1,\cdots ,q;j=1,\cdots ,u;\;\left(j\neq k\right)$$

where *s.t.* denotes the set of constraints, and p indicates the efficiency value of the research unit. When p < 1, the DMU is invalid in the model, indicating that it has deviated from the PF, and the land-resource-use efficiency is low. Theoretically, improvement is based on reducing input and increasing output, making it possible to intensively use of land resources. When p ≥ 1, the DMU is valid in the model, showing that it is at the PF, that is, the point of production efficiency. A larger p represents higher efficiency; *λ* represents the proportion of a DMU that is reassembled in a new effective DMU; *a_ij_* represents the *i*-th input of the research unit *j*; *b_tj_* is the *t*-th output of the research unit *j*; *k* is the DMU; *e* is the number of input indicators; *r*_1_ and *r*_2_ represent the numbers of expected and unexpected output factors, respectively; and s represents the slack variable of the input–output factors. Ferrier and Lovell believe that slack variables can ultimately be regarded as invalid resource allocation [45]. Among them, *s*^−^ is the input slack, which indicates the excess of input elements in the DMU, and *s^g^* is the expected output slack, which indicates the output of the DMU is insufficient. Finally, *s^h^* is the undesired output slack, which represents the surplus of the output factors of the DMU. 

#### 2.3.4. Malmquist–Luenberger Model

When the data of the evaluated DMU are panel data containing observations at multiple time points, the variation of productivity and decomposition factors of the variation can be analysed. The Malmquist model index is a non-parametric method commonly used to dynamically analyse changes in productivity. The Malmquist index model that includes undesired output is called the Malmquist–Luenberger (ML) model and is used to measure total factor productivity (TFP) with undesired output. It is also known as the ML index. It takes into account the intertemporal effect of dynamic factors and is strong in terms of practical applications [46]. In addition, compared to parametric methods, it has the following advantages: First, it does not need to provide the specific statistical distribution of the DMU. Second, it can deal with small amounts of data and classification variables. Third, it does not need to introduce a temporal trend into the data analysis, helping to avoid the phenomenon of smooth productivity change, which is an issue with most parametric methods [47]. The ML index is favoured by scholars based on these advantages.

Therefore, to study the evolution mechanism of dynamic efficiency, this study referred to the improved method of Fare [48,49] to calculate the ML index as follows:

(7) $$ML\left(x^{t},y^{t},x^{t+1},y^{t+1}\right)=\sqrt{\frac{E^{t}\left(x^{t+1},y^{t+1}\right)\;E^{t+1}\left(x^{t+1},y^{t+1}\right)}{E^{t}\left(x^{t},y^{t}\right)\;E^{t+1}\left(x^{t},y^{t}\right)}}$$

(8) $$=\frac{E^{t+1}\left(x^{t+1},y^{t+1}\right)}{E^{t}\left(x^{t},y^{t}\right)}\sqrt{\frac{E^{t}\left(x^{t},y^{t}\right)\;E^{t}\left(x^{t+1},y^{t+1}\right)}{E^{t+1}\left(x^{t},y^{t}\right)\;E^{t+1}\left(x^{t+1},y^{t+1}\right)}}$$

(9) =EC∗TC

where $\left(x^{t},y^{t}\right)\;$is the input–output vector in the period t; $\left(x^{t+1},y^{t+1}\right)$ is the input–output vector in the period t+1; and $E^{t}$ and $E^{t+1}$ represent the distance function during t and t + 1. The ML index is used to measure the changes in construction land-use efficiency, and it expresses the productivity change of $\left(x^{t+1},y^{t+1}\right)$ relative to $\left(x^{t},y^{t}\right)$. If ML > 1, the productivity level increases; otherwise, it decreases. The ML can be split into two aspects to obtain two decomposition indices: the technological change (TC) and technical efficiency change (EC). The TC index reflects the contribution of technological progress, such as system-element optimisation and economic structural transformation, to improve construction land-utilisation efficiency [50]. If the value is greater than 1, the production technology has improved. The EC index reflects the distance of the evaluation unit relative to the PF in different periods and is called the ”catch-up effect”. When its value is greater than 1, this indicates progress in technical efficiency, meaning that the allocation of construction land input resources is reasonable, and the management level has improved [51].

#### 2.3.5. Entropy-Weight TOPSIS Model

The entropy-weight TOPSIS model is derived from combining the entropy-weight and TOPSIS methods. Overall, it is a novel comprehensive evaluation method that scholars use to combine the advantages of the two methods and overcome the subjectivity of the index-weight setting [52]. The model has the advantages of less data loss in the calculation process, intuitive geometric meaning, and a lack of interference by the selection of reference sequences. It is also able to reflect the dynamic changes and laws of the evaluation indicators more scientifically, objectively, comprehensively, and reasonably, allowing a better explanation of the results [53]. Therefore, this study used this method to integrate the sub-indices that cover construction land-use inputs and outputs and pollution-emission levels. The calculation steps are as follows [54]:

First, the dimensional difference of measure index, $S_{it}$, was eliminated through standardisation as follows:

(10) $$S_{it}=\frac{M_{it}-min\left(M_{it}\right)}{max\left(M_{it}\right)-min\left(M_{it}\right)},M_{it}\;\mathrm{is}\;a\;\mathrm{positive}\;\mathrm{indicator}$$

Here, *i* represents the study area, $M_{it}$ refers to the initial value of the indicator and $S_{it}\;$is the standardised value.

Second, *j* is a sub-index. By calculating the information entropy $e_{j}$ and weight $W_{j}$ of $S_{it}$, a weighting matrix *R* is constructed as follows:

(11) $$e_{j}=\ln\frac{1}{x}\sum_{i=1}^{x}[\left(S_{it}/\sum_{i=1}^{x}S_{it})\ln(S_{it}/\sum_{i=1}^{x}S_{it}\right)]$$

(12) $$W_{j}=\left(1-e_{j}\right)/\sum_{j=1}^{m}\left(1-e_{j}\right)$$

(13) $$R={\left(a_{ij}\right)}_{x\times m,\;}a_{ij}=W_{j}\times S_{ij}$$

Again, we determine the optimal scheme $G_{t}^{+}$, the worst scheme $G_{t}^{-},$ and the corresponding Euclidean distances $h_{i}^{+}$ and $h_{i}^{-}$ as follows:

(14) $$G_{t}^{+}=\left(\max a_{i1},\max a_{i2},\cdots ,\max a_{im}\;\right)$$

(15) $$G_{t}^{-}=\left(\min a_{i1},\min a_{i2},\cdots ,\min a_{im}\right)$$

(16) $$h_{i}^{+}=\sqrt{\sum_{j=1}^{m}{\left(G_{j}^{+}-a_{ij}\right)}^{2}}$$

(17) $$h_{i}^{-}=\sqrt{\sum_{j=1}^{m}{\left(G_{j}^{-}-a_{ij}\right)}^{2}}$$

Finally, the comprehensive index *K* of the evaluation object is calculated, and its value range is (0, 1):

(18) K=hi−hi++hi−

## 3. Results

### 3.1. Static Analysis of Urban Construction Land-Utilisation Efficiency

**Figure 3:**
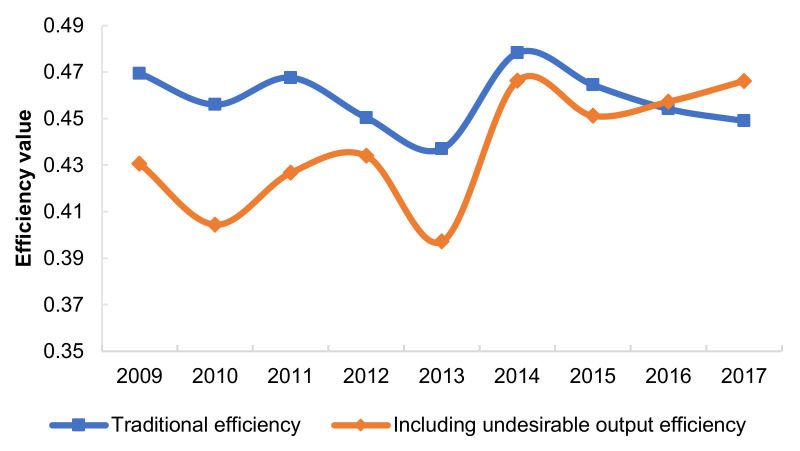
Average efficiency of construction land in the Yangtze River Delta from 2009 to 2017.

To obtain a more comprehensive understanding of the land-use situation in the Yangtze River Delta, this study used the MaxDEA(Beijing Rewomaidi Software Co., LTD, Beijing, China) professional software to calculate two types of construction land-utilisation efficiency in 41 prefecture-level cities in the Yangtze River Delta from 2009 to 2017. The results are shown below (Figure 3).

In general, the average construction land-use efficiency in the Yangtze River Delta from 2009 to 2017 was low, and the overall efficiency under the influence of pollution emissions (0.397–0.466) was slightly lower than the traditional efficiency (0.437–0.478). Although the efficiency dropped by 4.67% in total, over time, two types of efficiencies exhibited a fluctuating growth trend, and the gap between them gradually narrowed. Therefore, although the inclusion of undesired outputs will reduce the efficiency value, it is able to reflect the actual situation of regional construction land utilisation more scientifically. Recently, the Yangtze River Delta region has shown results based on the implementation of an ecological civilisation strategy, transforming the economic development mode, conserving energy, and reducing emissions, which has caused the construction land efficiency, including undesired outputs, to catch up with the traditional efficiency. However, overall, the low efficiency level reflects the current regional land-use pattern and is relatively extensive, and the land output is far from optimal. This is not conducive to promoting new urbanisation and high-quality city development, and there is room for the efficiency to be improved.

**Figure 4:**
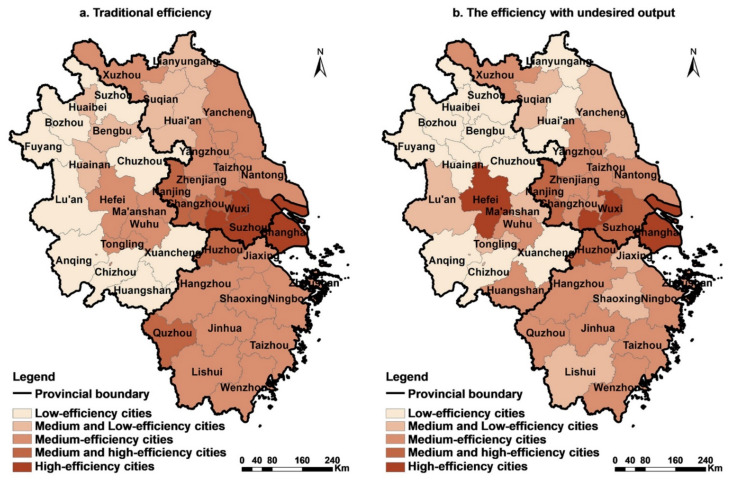
Mean value comparison between the traditional efficiency of urban construction land and the efficiency with undesired outputs in the Yangtze River Delta from 2009 to 2017. (**a**) Traditional efficiency; (**b**) efficiency with undesired outputs.

At the city level, traditional efficiency presents regional differentiation, being higher in the east and lower in the west (Figure 4a). Cities with high construction land efficiency are mostly located in the Jiangsu, Zhejiang, and Shanghai regions, which have significant geographical advantages, a high level of urbanisation, and developed economies. The Anhui region, which is located in a remote location in the Yangtze River Delta, has a relatively undeveloped economy. Therefore, coupled with insufficient radiation from the metropolitan area and core cities, the land-use efficiency of cities in this province is generally low. However, if environmental constraints are considered, this regional difference will be decreased (Figure 4b).

To better understand the constraints of undesired outputs, this study comprehensively analysed the impact of environmental factors on land-use efficiency based on the difference between the two efficiencies and the actual pollution-emission intensity of the city. Tests using the Stata16 software and an ordinary least squares (OLS) regression analysis method revealed a significant positive correlation between the efficiency differences and pollution emissions (t = 2.29; *p* < 0.05; where t is the regression coefficient and *p* is significance level), which indicates that the higher the pollution-emission intensity of a city, the greater the efficiency loss caused by undesired outputs.

(1) Southern Jiangsu, northern Zhejiang, and south-eastern Anhui have experienced greater efficiency losses (>0.1) due to a high pollution intensity. For example, Suzhou has been reduced from a high-efficiency city to a medium-high-efficiency city due to its pollution intensity of 0.192. Furthermore, six cities, namely, Jiaxing, Shaoxing, Lishui, Ma’anshan, Wuhu, and Tongling, are affected by high pollution emissions, and their land-use efficiency has changed from medium to low.

(2) The construction land utilisation efficiency of eastern Zhejiang, northern Jiangsu, and northern Anhui was moderately negatively affected by undesired outputs (0.05–0.1) due to a higher pollution intensity. Amongst these regions, the land-use efficiency of Changzhou and Quzhou changed from medium-high to medium, and that of Yancheng changed from medium to medium-low. The efficiency of the four cities of Huai’an, Lianyungang, Huaibei, and Huainan were originally at the lower-middle level. Due to the impact of pollution emissions, the efficiency was further reduced.

(3) The difference between the two efficiencies is relatively small in most cities in southwestern Anhui due to the low total pollution emissions. For example, the pollution-emission indices of some cities, such as Wuhu and Anqing, are less than 0.008, which results in a small degree of efficiency loss. Therefore, the real efficiency of several high-efficiency cities in the region is greatly reduced due to exorbitant environmental pollution emissions, whereas some low-efficiency cities are less affected by relatively small emissions of pollution, and their efficiency only fluctuates within a small range. Accordingly, the efficiency differences within the region are reduced, and the spatial equilibrium of the efficiency is strengthened. 

### 3.2. Analysis of the Driving Force of the Evolution of Urban Construction Land-Utilisation Efficiency

To further realise dynamic changes in urban construction land-utilisation efficiency and their driving factors, this study measured the ML and its decomposition index (TC and EC) and analysed these accordingly.

**Figure 5:**
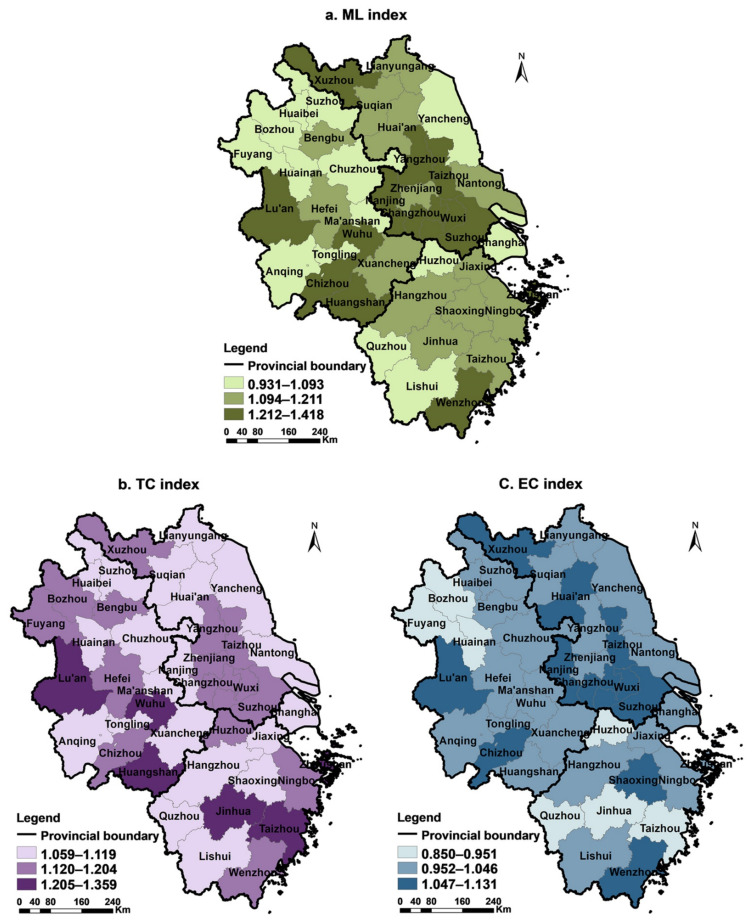
Annual average values of ML, TC, and EC of urban construction land in the Yangtze River Delta. (**a**) The spatial distribution of ML index; (**b**) spatial distribution of TC index; (**c**) spatial distribution of EC index.

Using the ArcMap10.6 software and Natural Breaks (jenks) method, the annual average ML, TC, and EC indices of 41 prefecture-level cities in the Yangtze River Delta were divided into three levels, from small to large (Figure 5). Generally, the ML of the Yangtze River Delta had the highest spatial distribution characteristics in southern Jiangsu and southern Anhui, followed by northern Zhejiang and northern Jiangsu. Lower spatial distribution characteristics were present in Shanghai, southern Zhejiang, and northern Anhui.

(1) As the largest cities in the region, Suzhou, Wuxi, and Changzhou, among others, have gathered much high-level talent due to their advantages regarding their locations for transportation, economic and technological development, and strong capital strength. These drive TC and EC towards improving regional industrial innovation and management system optimisation (TC > 1.12; EC > 1.05). This has resulted in a significant improvement in the ML (1.418 > ML > 1.212), and the efficiency of construction land increased significantly during the study period. Recently, Lu’an, Huangshan, and Chizhou have reorganised their input production factors, optimised resource allocation, and strengthened their technological updates and creations to promote the rapid growth of ML. 

(2) The economic strengths of northern Zhejiang and northern Jiangsu are relatively lower than those of other regions. Although they have certain technological advancement capabilities, such as research and development (R&D) investment and technological output (1.12 < TC < 1.20), some cities are hindered regarding the improvement of ML due to a low EC (EC < 1), such as an imbalanced allocation of input resources or rigid management systems (1.094 < ML < 1.211). Additionally, though the efficiency of this type of urban construction land has improved, there is still plenty of room for improvement in the future. As a large city in the Yangtze River Delta, Shanghai has unique advantages in science, technology, policy, information, and capital. However, its land-use efficiency has reached the highest level on the common frontier, and there is limited room for its efficiency to improve. Therefore, its ML is low, but its efficiency is still in a slow growth status. 

(3) The cities of Quzhou, Lishui, Huaibei, Suzhou, and Chuzhou in southern Zhejiang and northern Anhui have relatively little advantage regarding technology application and achievement conversion, and their technological progress is not obvious (1 < TC < 1.09). Coupled with their inadequacies regarding resource allocation and information circulation, they are restricted by technology efficiency (EC < 1), which results in little improvement in their ML and a lack of potential for the improvement of urban construction land efficiency.

In general, the average ML value for construction land in the Yangtze River Delta is 1.154, the TC index is 1.142, and the EC index is 1.010. The values of the three indicators are all greater than one, which indicates that the improvement of ML is affected by the combined effect of the TC and EC. However, the contribution rate for TC is 14 times that for EC. In addition, the average value of each index of the above case city shows that 98% of the cities’ ML indices are greater than 1, and 41 cities are driven by TC, whereas only 63.41% of the cities are driven by EC. Moreover, the growth of the ML indices of the other 36.59% of the cities is restrained due to a low EC. Therefore, TC is the core driver for the improvement of ML, and the contribution of EC is relatively insignificant.

### 3.3. Correlation Analysis of Urban Construction Land-Utilisation Efficiency and Factor Inputs and Outputs

Using the method presented by Liu [55], the element input and output and pollution-emission levels during the construction land-utilisation process were analysed to further determine the internal causes of the differentiation in regional efficiency. As the factor input index, the unit land labour force and fixed-assets investment represent the land input level. Using the land-average production value as the expected output index for land use indicates the level of land output. Moreover, the land-average wastewater, sulphur dioxide, and dust emissions were used as undesired output indicators of land use to characterise the degree of land-use pollution emissions. In addition, using the entropy-weight TOPSIS method, three kinds of indicators of the input, expected output, and unexpected output were, respectively constructed into three kinds of comprehensive indices of input, expected output, and non-expected output, to scientifically and comprehensively measure the input–output situation of land. Finally, the K-means clustering method was used to divide the comprehensive indicators of construction land-use input and output and pollution emissions into the following three types: low, medium, and high (Table 2). 

(1) Generally, a high input and output will result in high efficiency. Shanghai’s land input and economic output are leading in the region, and its construction land utilisation maintained a high level of efficiency during the study period. Moreover, a low input and output often result in low efficiency. Cities with low input, output, and pollution comprise more than 46% of the Yangtze River Delta region, and most of them are located in marginal areas of northern Jiangsu, northern Zhejiang, northern Anhui, and southwest Anhui, such as Lianyungang, Huaian, Lishui, and Chizhou. Such cities are relatively deficient in economic and technological development and resource management and allocation, which results in insufficient input and a low output. Although the pollution degree is small, as the actual output of construction land at this time has become the fundamental constraint for efficiency evaluation, the land-use efficiency is low. With the transformation of the economic structure and the continuous progress of society, some areas, such as Yangzhou and Wenzhou, have gradually shifted from low input to medium-high input, while the land-use output has risen, and the efficiency of construction land has increased.

(2) However, during the process of urban development, the utilisation efficiency for construction land is not always consistent with the level of land input and output due to improper resource allocation or pollution discharge. Although Changzhou, Nanjing, Wuxi, Ma’anshan, and Wuhu, among other cities, have high input levels, they also have redundant or unreasonable input elements. Therefore, the land output failed to achieve synchronous growth. At the same time, these areas experience medium to high levels of pollution emissions during the process of economic construction, which further reduces the efficiency of land use. In addition, low and medium inputs may result in a higher land output and land-use efficiency on the basis of controlling pollution emissions. In 2009, cities such as Huzhou, Jinhua, and Quzhou adopted low input, medium output, and low pollution land use model to achieve a relatively high level of construction land use efficiency. In 2017, some cities, such as Hefei and Nanjing, adopted a medium input and output and reduced the total pollution emissions to make more efficient use of construction land. However, although Hangzhou’s land input and output levels are relatively high, its construction land efficiency is always in the lower position for the same level of cities due to negative impacts, such as high pollution emissions.

(3) In addition, medium inputs and low outputs will be the top priority of land-market-consolidation efforts in the future. The associated cities are mainly located in central Jiangsu and other areas with good economic development. They have strong comprehensive strength and development potential and can provide sufficient input for land use. However, due to their limitations, including technical conditions and inadequate means of resource allocation, the improvement of land efficiency has encountered bottlenecks, resulting in a clear deviation of land use from the optimal PF. In the future, we should improve the quality and efficiency of land use through comprehensive land consolidation and remould the land-use pattern.

## 4. Discussion

Urban construction land resources have become an important bottleneck restricting urban economic and social development. China’s strict farmland-protection policies have significantly blocked the external supply of construction land resources [56]. In the context of new urbanization, each region should fully consider its own resource endowments and functional positioning, relying on the new era of territorial space planning, and comprehensively improve the use efficiency for urban construction land. The research of relevant scholars shows that the impact and rate of contribution of construction land expansion on economic growth gradually decreases with the evolution of economic development [57]. The development of construction land will play a more obvious driving economic role in underdeveloped areas [58]. The Yangtze River Delta region is one of the most economically developed regions in China, and the southern Jiangsu and northern Zhejiang and Shanghai areas have entered the later stages of urbanization. Therefore, these cities should pay attention to the connotative development of the city, supplement them with territorial space planning, and tap the potential of the urban stock land through the reconstruction of the old city, urban village renewal, and the development of idle and inefficient land. As northern Jiangsu, northern Anhui, and western Zhejiang are in the middle and later stages of urbanization, with the advancement of China’s common prosperity, these areas need more indicators of construction land to drive economic development, which requires the overall coordination of construction land resource allocation in the compilation of territorial space planning. In view of the excellent ecological endowments in northern Jiangsu, northern Anhui, and western Zhejiang, the construction land index should be appropriately tilted to them under the conditions that the regional resource and environmental carrying capacity permit. Central Jiangsu, southern Anhui, and southern Zhejiang should give full play to their regional advantages, adjust the industrial structure to attract the transfer of industries in the core area of the Yangtze River Delta, and increase the level of land output. 

Generally speaking, the economic development of certain regions is often accompanied by the excessive use of resources and greenhouse gas emissions. Given that the overall construction land-use efficiency in the Yangtze River Delta under environmental constraints is low, the regional imbalance is prominent, and the improvement of efficiency mainly comes from TC, we believe that further targeted efficiency-improvement measures should be taken in future. First, we should assume that the political responsibility and development mission of “putting ecology and green development first” strengthens the concept of ecological protection. Then, we should jointly build a strong environmental constraint mechanism and implement the negative list system for industrial access. In areas where the input and output levels are high, but the efficiency is reduced due to high pollution emissions, it is necessary to pay special attention to ecological protection and enhance the competitiveness of green development. Second, the Yangtze River Delta should prioritize the powerful driving effect of provincial capitals or large cities, promote overall regional efficiency, and strive to create high-quality integrated regional developmental growth. Third, the exchange culture of sharing resources and win–win cooperation should be promoted. By strengthening inter-regional communal work and rationally scheduling the spatial transfer and allocation of product factors between cities, the Yangtze River Delta region can overcome the contradiction between the production scale and technical structure that exists in economic, social, and environmental activities in various regions. With institutional innovation as the core, using policy innovation as the key factor and technological innovation as the driving force (based on continuous technological advancement), the Yangtze River Delta region can further improve EC and the technology catch-up effect by optimizing the scale allocation and management level of construction land input resources. Accordingly, the use efficiency of construction land will be evenly increased.

This paper has several shortcomings. First, due to the limited data acquisition regarding the measurement of urban construction land efficiency that includes undesired outputs, the total emissions made up of only three types of waste discharge were selected as environmental constraints to represent the level of urban pollution emissions. Follow-up studies could consider deepening and perfecting the evaluation indicators of undesired outputs in terms of environmental protection investment, carbon emissions, environmental governance, and air quality (PM_2.5_). Second, this study used only 41 prefecture-level cities in the Yangtze River Delta as its research object. If county-level cities could be selected for research in the future, the efficiency of regional construction land use would be reflected more comprehensively and accurately. Finally, although this study performed a correlation analysis for the efficiency of urban construction land use, input factors, and output levels, it did not explore specific input and output types in depth, which must be expanded upon in future research.

## 5. Conclusions

Based on the land input–output data for 41 prefecture-level cities in the Yangtze River Delta, this study constructed an SE-SBM model to compare and evaluate the static efficiency of urban construction land use from 2009 to 2017 considering traditional and environmental constraints. Furthermore, using the ML index and its decomposition items, the dynamic evolution of the construction land-use efficiency was studied. In addition, the entropy-weight TOPSIS method was used to analyse the relationship between the urban construction land efficiency and input–output level. The research conclusions are as follows:

(1) Based on the results of the static efficiency measurement, it was found that the construction land efficiency of the Yangtze River Delta region remained at a low level, overall, during 2009–2017. Furthermore, due to the addition of undesired outputs, the efficiency dropped by 4.67%. However, with the transformation and upgrading of the economy structure, the land-use efficiency under environmental constraints has gradually caught up with the traditional efficiency, and the total efficiency has increased slightly. At the city level, the traditional construction land efficiency presents regional differentiation, being higher in the east and lower in the west. High-efficiency cities are concentrated in the economically developed Jiangsu, Zhejiang, and Shanghai regions, and most of the low-efficiency cities are located in the economically underdeveloped Anhui region. Due to the influence of pollution emissions, the regional imbalance in efficiency has been weakened. This change stems from the efficiency loss caused by pollution emissions, and the degree of efficiency loss is significantly positively correlated with the intensity of urban pollution emissions. For example, southern Jiangsu, northern Zhejiang, and south-eastern Anhui have lost more than 0.1 efficiency due to their high pollution emission intensity, and eastern Zhejiang, northern Jiangsu, and northern Anhui suffered 0.05–0.1 efficiency losses due to their medium-to-high pollution emission intensity. Cities in southwest Anhui only lost small amounts of efficiency due to their lower pollution emissions. Compared to the traditional-efficiency values, the gap between high and low-efficiency cities in the region has been narrowed, decreasing the spatial differentiation of efficiency.

(2) Based on the results of the dynamic efficiency measurements, it was found that the ML in southern Jiangsu and southern Anhui has the fastest growth, followed by northern Zhejiang and northern Jiangsu, while the growth rates of Shanghai, southern Zhejiang, and northern Anhui are relatively slow. Amongst them, the ML productivity of construction land in 98% of cities is mainly improved by TC, whereas EC has a limited promoting effect and does not cover the whole area. Therefore, although, during the process of construction land use for production, the economic structure of most regions has been transformed and upgraded, the institutional system has been optimized, and scientific and technological innovations have been iterated, small and medium-sized cities still have outstanding problems. These include a lack of ecological protection, an unbalanced allocation of resource elements, unreasonable investment scales, and low management levels, restricting the further improvement of construction land-use efficiency and exacerbating regional differentiation.

(3) Using the entropy-weight TOPSIS model and K-means clustering method, the construction land input and output and pollution emission indicators of 41 cities in the Yangtze River Delta were divided into the following three types: low, medium, and high. The correlation between the efficiency and the input and output was then investigated. The results show that, under normal circumstances, there is a correlation between construction land-use efficiency and land input and output; namely, a high input and output lead to high efficiency, and a low input and output lead to low efficiency. However, the two are not always consistent. A medium input and output and a low input and medium output may result in higher land-use efficiency resulting from the control of pollution emissions. Medium-input and low-output types have sufficient room for efficiency improvement and should be the top priority in comprehensive land consolidation in the future.

## Figures and Tables

**Table 1:** Input–output indicator system for urban construction land-utilisation efficiency.

Indicator	Type	Index Content
Input indicators	Land	Urban construction land area/hectare
	Capital	Fixed-asset investment in the whole society/100 million yuan
	Labour force	Employees in the secondary and tertiary industries/ten thousand people
Output indicators	Expected output	GDP/100 million yuan
	Undesired output	Industrial wastewater discharge/ton
		Industrial sulphur dioxide emissions/ton
		Industrial smoke and dust emissions/ton

**Table 2:** Correlation types for input–output and pollution emissions of construction land use in cities of the Yangtze River Delta.

Input–Output Model of Construction Land	2009	2017
	City	City
High input, high output, low pollution	Shanghai (1.299)	/
High input, medium output, high pollution	Changzhou (0.369)	/
High input, medium output, medium pollution	Nanjing (0.445), Wuxi (0.592), Ma’anshan (0.366)	/
High input, medium output, low pollution	Hefei (1.234)	Wenzhou (1.004)
High input, low output, low pollution	Wuhu (0.301)	Zhoushan (0.462)
Medium input, high output, medium pollution	/	Shanghai (1.291)
Medium input, medium output, high pollution	Hangzhou (0.439)	/
Medium input, medium output, medium pollution	Suzhou (0.637)	Wuxi (1.04), Changzhou (0.721), Hangzhou (0.52)
Medium input, medium output, low pollution	Ningbo (0.549), Zhoushan (0.436)	Nanjing (1.006), Ningbo (0.525), Hefei (1.032)
Medium input, low output, high pollution	/	Shaoxing (0.366)
Medium input, low output, medium pollution	Shaoxing (0.206)	Jiaxing (0.323), Ma’anshan (0.28)
Medium input, low output, low pollution	Nantong (0.355), Zhenjiang (0.454) Taizhou (0.315), Jiaxing (0.226), Xuancheng (0.157), Tongling (0.265)	Yangzhou (0.494), Zhenjiang (0.524), Taizhou (0.62), Wuhu (0.399), Tongling (0.261)
Low input, medium output, medium pollution	Quzhou (1.044)	Suzhou (1.016)
Low input, medium output, low pollution	Huzhou (1.03), Jinhua (1.025), Taizhou (1.011)	Xuzhou (1.089)
Low input, low output, medium pollution	/	Quzhou (0.272)
Low input, low output, low pollution	Xuzhou (0.373), Lianyungang (0.229) Huaian (0.198), Yancheng (0.315), Yangzhou (0.379), Suqian (0.19), Wenzhou (0.46), Lishui (0.303), Huaibei (0.254), Bozhou (0.239), Suzhou (0.237), Bengbu (0.238), Fuyang (0.279), Huainan (0.263), Chuzhou (0.172), Lu’an (0.216), Chizhou (0.164), Anqing (0.203), Huangshan (0.189)	Nantong (0.445), Lianyungang (0.283), Huaian (0.381), Yancheng (0.289), Suqian (0.268), Huzhou (0.306), Jinhua (0.362), Taizhou (0.421), Lishui(0.288), Huaibei (0.229), Bozhou (0.16), Suzhou (0.177), Bengbu (0.276), Fuyang (0.135), Huainan (0.164), Chuzhou (0.188), Lu’an (0.422), Xuancheng (0.2), Chizhou (0.419), Anqing (0.192), Huangshan (0.265)

## Data Availability

All the socio-economic data can be found in the China statistical Yearbook, and the land data can be found in the Ministry of Natural Resources. All the socio-economic data can be found here: https://data.cnki.net/Yearbook/Navi?type=type&code=A. The land data are not public.
